# Heterotetrameric annexin A2/S100A10 (A2t) is essential for oncogenic human papillomavirus trafficking and capsid disassembly, and protects virions from lysosomal degradation

**DOI:** 10.1038/s41598-018-30051-2

**Published:** 2018-08-03

**Authors:** Julia R. Taylor, Daniel J. Fernandez, Shantaé M. Thornton, Joseph G. Skeate, Kim P. Lühen, Diane M. Da Silva, Ralf Langen, W. Martin Kast

**Affiliations:** 1 Department of Molecular Microbiology & Immunology, University of Southern California, Los Angeles, CA USA; 2 Department of Obstetrics & Gynecology, University of Southern California, Los Angeles, CA USA; 3 Norris Comprehensive Cancer Center, University of Southern California, Los Angeles, CA USA; 4 Department of Biochemistry and Molecular Biology, University of Southern California, Los Angeles, CA USA

## Abstract

Human papillomavirus (HPV) entry into epithelial cells is independent of canonical endocytic pathways. Upon interaction with host cells, HPV establishes infection by traversing through an endocytic pathway that is clathrin- and caveolin-independent, but dependent on the annexin A2/S100A10 heterotetramer (A2t). We examined the contribution of monomeric annexin A2 (AnxA2) vs. A2t in HPV infection and endocytosis, and further characterized the role of these molecules in protein trafficking. We specifically show that cell surface A2t is not required for HPV attachment, and in the absence of A2t virion internalization remains clathrin-independent. Without A2t, viral progression from early endosomes to multivesicular endosomes is significantly inhibited, capsid uncoating is dramatically reduced, and lysosomal degradation of HPV is accelerated. Furthermore, we present evidence that AnxA2 forms a complex with CD63, a known mediator of HPV trafficking. Overall, the observed reduction in infection is less significant in the absence of S100A10 alone compared to full A2t, supporting an independent role for monomeric AnxA2. More broadly, we show that successful infection by multiple oncogenic HPV types is dependent on A2t. These findings suggest that A2t is a central mediator of high-risk HPV intracellular trafficking post-entry and pre-viral uncoating.

## Introduction

Persistent infection with mucosal-tropic high-risk human papillomavirus (HPV) causes cervical, vaginal, anal, penile, and oropharyngeal cancers^1–3^. HPV-associated diseases inflict a significant disease burden on the global population, yet there remain unanswered questions about HPV cellular entry. As such, initial establishment of HPV infection remains an active area of investigation. HPV is a non-enveloped double-stranded DNA virus composed of major capsid protein L1 (HPV L1) and minor capsid protein L2 (HPV L2)^4^. Although structurally simple, HPV infection depends on the exploitation of complex host cell machinery and endocytic processes. HPV type 16 (HPV16) is the most common oncogenic genotype and is widely used to study the infectious lifecycle of HPV. Since 1995, HPV entry has been thought to be receptor-mediated; nevertheless, a consensus HPV receptor has still not been identified^5^. Although many HPV entry-associated molecules and co-factors have been recognized in what is shaping up to be an incredibly complex and unique endocytic pathway (recently reviewed in^6^), a central mediator has yet to be described.

The literature to date has shown that HPV16 endocytosis into host basal epithelial cells is independent of canonical clathrin-, caveolin-, flotillin-, lipid raft-, cholesterol-, and dynamin-mediated endocytosis^7–9^. Trafficking of HPV from the cell surface to the nucleus can be broken down into five key stages: cell surface binding, entry, viral vesicle trafficking, capsid uncoating, and transporting of the viral genome (vDNA) through the trans-Golgi network (TGN) to the nucleus. HPV binds to the cell surface through two distinct attachment events. First, HPV capsid proteins interact with heparan sulfate proteoglycans (HSPGs) found on the plasma membrane of basal keratinocytes or within the surrounding extracellular matrix^10–13^. The binding of HPV to HSPGs induces conformational changes in both HPV L1 and L2^14–16^, exposing the amino terminus of HPV L2 which contains a furin/proprotein convertase cleavage site^17^. These conformational changes in the capsid reduce HSPG-affinity and the virion is then transferred to the elusive secondary uptake receptor/receptor complex located within tetraspanin enriched microdomains (TEMs)^9,18,19^. Candidate receptors to date have included α6 integrin^20,21^, epidermal growth factor receptor^22,23^, and the protein complex studied herein – the annexin A2 heterotetramer (A2t)^24,25^. After handoff to this secondary receptor/receptor complex, HPV is internalized through a non-canonical endocytic mechanism and trafficked through the degradative endosomal system. While it has been shown that in optimal conditions viral trafficking may be rapid, bulk internalization is relatively slow and asynchronous due to the time it takes for extracellular structural modifications of the capsid and a hypothesized limited availability of the secondary receptor/receptor complex^26,27^. Internal trafficking is dependent on endocytic mediators including, but not limited to Rab GTPases, certain components of the ESCRT machinery, sorting nexin 17, and the cytoskeletal adapter protein obscurin-like 1 protein (OBSL1)^8,28–31^. Through this process, early HPV-containing endosomes are delivered to multivesicular endosomes (MVEs) where the majority of capsid uncoating occurs through compartment acidification and cyclophilin-mediated dissociation of the viral genome (vDNA) and capsomeres^32,33^. Delivery of HPV to MVEs is dependent on CD63, a tetraspanin that has been shown to facilitate HPV trafficking and directly interact with the viral capsid^34^. The vDNA, concealed within a vesicle, then escapes lysosomal degradation by transport to the TGN via interaction of cytosolically exposed HPV L2 with the retromer complex^35–37^. The vDNA-containing vesicle eventually infiltrates the nucleus during the nuclear envelope breakdown step of mitosis, completing intracellular trafficking and establishing infection^38,39^.

Previous evidence suggests a role for A2t at the cell surface and in the intracellular trafficking of HPV^24,25^. However, the function and necessity of A2t at the cell surface and the precise endocytic steps mediated by the heterotetramer and/or its individual subunits in HPV infection are not well understood. A2t is a multifunctional membrane-associated protein complex composed of two annexin A2 (AnxA2) monomers bridged by an S100A10 homodimer^40–42^. AnxA2 and S100A10 are expressed in many tissues, have been studied in the context of diverse cellular processes, and are linked to multiple aspects of human health and disease^43–45^. The AnxA2 monomer and A2t, however, have independent functions and should therefore be considered biochemically distinct. In the mucosa, A2t is expressed in the basal layer of the epithelium^46,47^, which is the initial site of HPV infection. On a cellular level, A2t is localized to the inner and outer leaflets of the plasma membrane, while AnxA2 can be found in the cytosol or membrane-bound. More specifically, these proteins have been implicated in membrane domain organization, membrane trafficking events, membrane-cytoskeletal connection through F-actin binding, MVE biogenesis, and membrane curvature^48–53^. S100A10 is a small dimeric helix-loop-helix protein found in close association with AnxA2 (complexed together to form A2t) and implicated in the trafficking of a variety of membrane-resident proteins^41^.

S100A10 is rapidly ubiquitinated and degraded in the absence of AnxA2^54,55^. A large proportion of studies investigating AnxA2 utilize anti-AnxA2 knock-down strategies, effectively removing both monomeric AnxA2 and heterotetrameric A2t. In the past, researchers have reported findings about either moiety without truly distinguishing between the two. Therefore, to resolve the ambiguity between AnxA2 and A2t in the context of HPV endocytosis, this study employs complete protein knockout of either S100A10 or AxnA2/S100A10 (A2t) in epithelial cells (HeLa) to distinguish the specific contributions of these host factors in HPV entry and intracellular trafficking. Here, we provide evidence supporting the role of A2t in HPV intracellular trafficking, and further characterize the involvement of S100A10 in protein trafficking. Our data suggest that AnxA2 and A2t are not required for cell surface attachment nor non-infectious internalization of HPV. Instead, these data reveal a role for A2t in post-entry intracellular trafficking and show that AnxA2 interacts with CD63, a known mediator of HPV trafficking. We show that in the absence of S100A10 alone or complete A2t, cellular entry of HPV is not reduced, however, progression of early HPV-containing endosomes to MVEs is significantly inhibited. Consequently, HPV capsid disassembly is repressed, and the virus is redirected to the lysosome for degradation. Taken together, these findings suggest that A2t is required for the infectious post-entry intracellular trafficking of HPV. Additionally, we reveal a previously underappreciated role for S100A10 in endosomal transport.

## Results

### Annexin A2 (AnxA2) and the annexin A2/S100A10 heterotetramer (A2t) are essential for the infection of high-risk oncogenic HPV in HeLa cells

**Figure 1:**
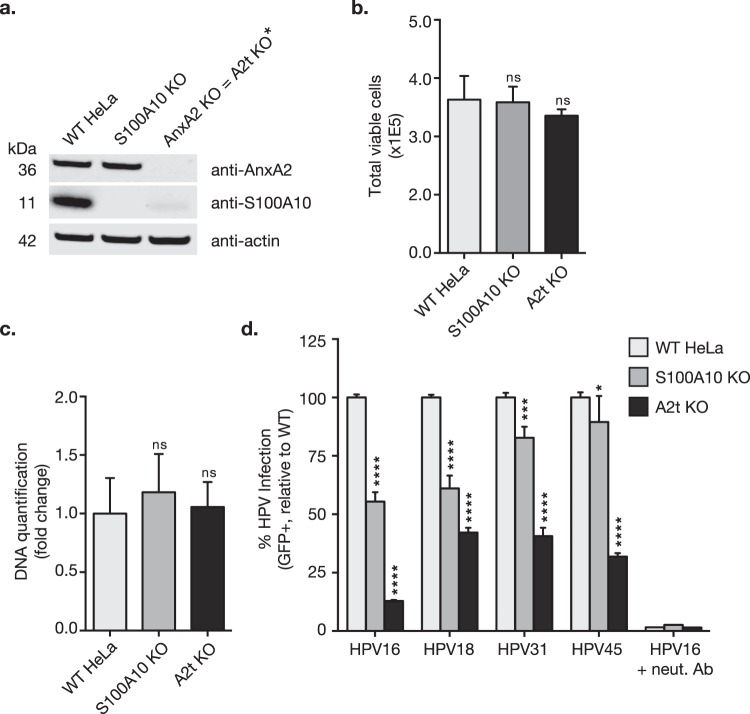
S100A10 knockout (KO) and AnxA2 KO (resulting in loss of full A2t) does not affect cell proliferation and inhibits multiple high-risk HPV infections in HeLa cells. **(a)** *Because S100A10 is rapidly degraded in the absence of AnxA2, *targeting AnxA2 results in full A2t KO*. Here, either dimeric S100A10 or heterotetrameric A2t (S100A10 + AnxA2) was knocked out in wild type (WT) HeLa cells via CRISPR/Cas9 and confirmed by western blot. Western blot images were cropped. See full-length blots with molecular weight standards in Supplementary Fig. 1a. **(b**,**c)** WT, S100A10 KO, and A2t KO HeLa cells were seeded with equal cell number, grown for 48 h, and then analyzed for differences in cell proliferation via **(b)** analyzing cell number and viability via trypan blue exclusion test, and **(c)** quantification of nucleic acid content via CyQUANT Cell Proliferation Assay Kit (Thermo Fisher) and then analyzed relative to WT HeLa cells. Results **(b**,**c)** represent N = 8 ± s.d. and are representative of at least 3 independent experiments. Statistics: 1-way ANOVA with Dunnett’s multiple comparison test. ns = not significant. **(d)** WT, S100A10 KO, and A2t KO HeLa cells were treated with HPV PsVs (TCID_50_) carrying a GFP reporter plasmid. Infection (GFP gene transduction) was measured 48 h p.i. via flow cytometry. Neutralizing anti-HPV L1 antibody H16.V5 (neut. Ab) was used as a positive control for infection inhibition, and background from mock infected cells was subtracted. At least 3 independent S100A10 KO and 3 independent A2t KO clones were screened for consistent inhibition of HPV16 infection. Results are representative of at least 3 independent experiments and show the mean %GFP-positive cells ± s.d. (n = 3, normalized to WT). Statistics: 1-way ANOVA with Dunnett’s multiple comparisons test – ns = not significant, *P < 0.05, ***P < 0.001, ****P < 0.0001.

We have previously shown that targeting A2t via siRNA knockdown of AnxA2, or targeting the AnxA2-S100A10 binding interface via small molecule inhibition significantly reduces HPV16 infection of cervical epithelial cells (HeLa and HaCaT)^24,56^. It has also been suggested that the A2t subunits, AnxA2 and S100A10, may play separable roles in HPV16 entry and intracellular trafficking^25^. Therefore, we sought to decipher the specific contributions of AnxA2 and A2t in HPV infection and endocytosis. To remove A2t, we knocked out either S100A10 or the full A2t complex via CRISPR/Cas9 gene editing and generated clonal HeLa cell populations. Given that S100A10 is rapidly degraded in the absence of AnxA2^54,55^, the AnxA2 knockout (KO) effectively serves as a full A2t subunit KO. Western blot analysis confirmed the absence of protein expression in the KO clones (Fig. 1a).

Because AnxA2 has been implicated in cytokinesis^57^, differences in cell proliferation between clonal populations and WT cells were analyzed to exclude altered proliferation rates as a potential confounding factor for the reduction in HPV infection that we observed. Cell counts and proliferation were measured via trypan blue exclusion and DNA-based quantification respectively (Fig. 1b,c), and no significant difference in proliferation was observed.

HPV gene transduction is indicative of successful viral entry, intracellular trafficking, and infiltration of the nucleus during mitosis; therefore, *in vitro* infection is defined as reporter gene transduction. Using HPV16 pseudovirions (PsVs) containing a GFP reporter plasmid, we then screened at least two S100A10 KO and two A2t KO clones and found consistent inhibition of infection (Fig. 1d), confirming previously reported findings^24,25^. To extend these findings and determine whether these proteins are used by other HPV genotypes, we tested additional high-risk oncogenic HPVs as previously A2t has only been implicated in HPV16 infection. Similar to HPV16, we found that infection by HPV18, -31, and -45 was also significantly inhibited in both KO cell lines (Fig. 1d), implicating this receptor complex in infection by multiple oncogenic HPVs.

### Restoration of A2t through annexin A2 knock-in restores HPV16 infectivity

**Figure 2:**
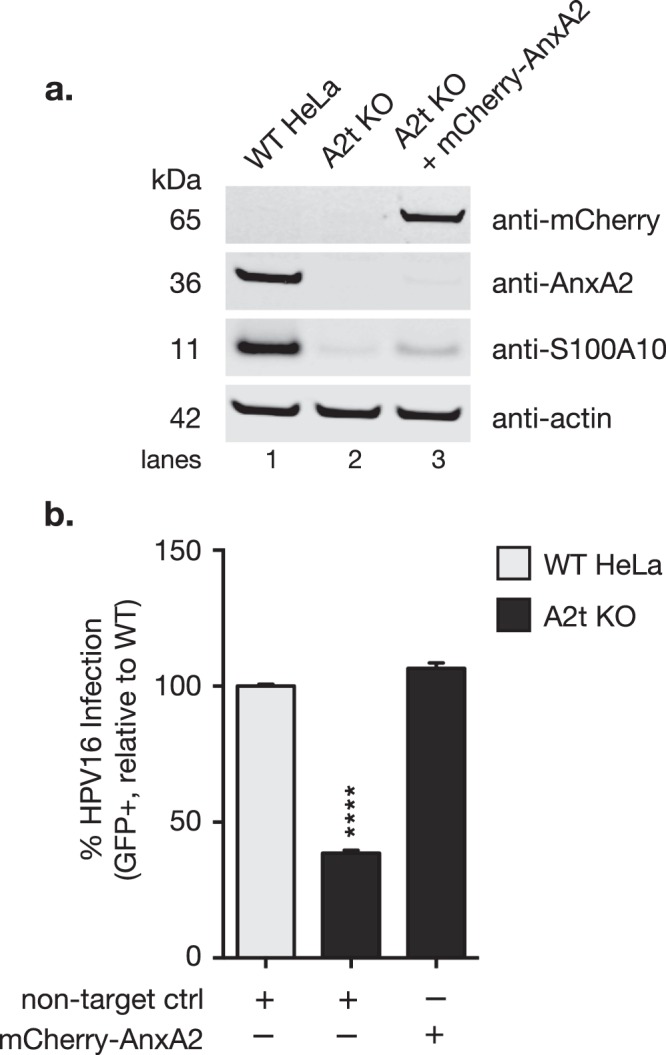
HPV16 infectivity is rescued by restoring A2t expression. **(a)** WT HeLa cells were transiently transfected with a control plasmid and A2t KO cells were transfected with either an mCherry-tagged AnxA2 expression plasmid or a non-target control plasmid. AnxA2 expression was restored 24 h post-transfection, stabilizing S100A10 and restoring heterotetrameric A2t (western blot, lane 3). Lysates were prepared with total populations of transfected cells, and mCherry-AnxA2 transfection efficiency was approximately 50%. Western blot images were cropped. See full-length blots with molecular weight standards in Supplementary Fig. 1b. **(b)** 24 h post-transfection, WT and A2t KO cells + non-targeting plasmid control and A2t KO cells + an mCherry-AnxA2 expression plasmid were treated with HPV pseudovirions (TCID_50_) carrying a GFP reporter plasmid. Infection was measured 48 h p.i. via flow cytometry, and infection rescue was measured by gating on mCherry and analyzing the %GFP-positive cells within that population. See mCherry gating strategy in Supplementary Fig. 2. Results show the mean %GFP-positive cells ± s.d. (n = 3, normalized to WT), and are representative of at least 3 independent experiments. Statistics: 1-way ANOVA with Dunnett’s multiple comparisons test – ****P < 0.0001.

To ensure that the observed inhibition of infection was specifically due to the S100A10 and AnxA2 gene editing, we restored AnxA2 expression in the A2t KO cells by plasmid transfection which stabilized S100A10 protein expression and thus reintroduced heterotetrameric A2t (Fig. 2a). Lane 3 in Fig. 2a represents mCherry-tagged AnxA2 (mCherry-AnxA2) expression rescue in a total cell population of A2t KO cells, where transfection efficiency was optimized to ~50% at 24 h post-transfection. Transfection efficiency was determined by transfecting cells and measuring %mCherry-positive cells 24 h post-transfection (Supplementary Fig. 2b). In order to analyze infection rescue in the mCherry-AnxA2-transfected cells, the labeled population was gated on mCherry (Supplementary Fig. 2c) and analyzed for %GFP-positive cells (Supplementary Fig. 2d). The data show that upon AnxA2 re-expression and A2t re-stabilization, infection was restored to WT levels (Fig. 2b), confirming that reduction in HPV16 infection in the A2t KO cells was not due to off-target effects.

### A2t is not required for HPV cell surface attachment

**Figure 3:**
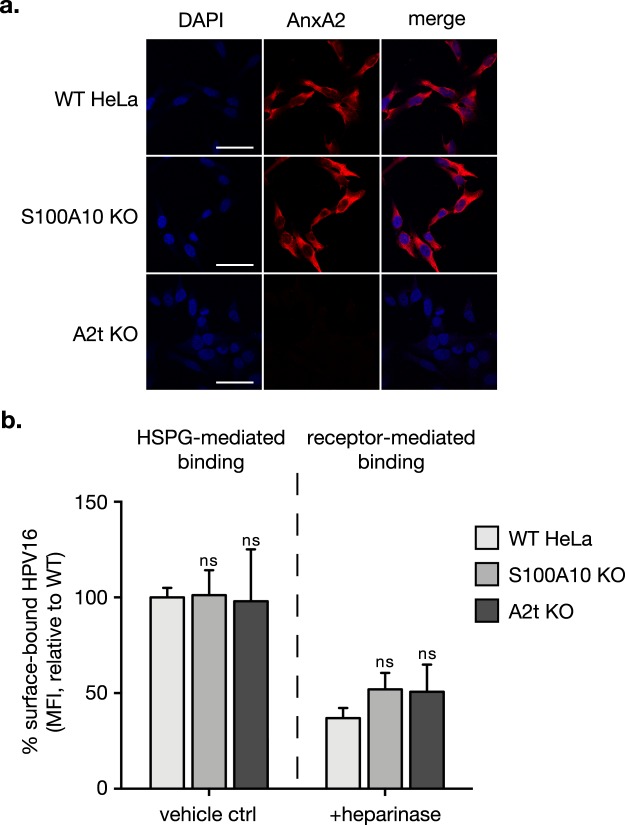
S100A10 and A2t are not required for cell surface binding of HPV16. HPV binding occurs through binding heparan sulfate proteoglycans (HSPG) and a receptor complex. **(a)** Wild type (WT) HeLa and S100A10 KO cells ubiquitously express AnxA2 at the cell surface. Here, cells were grown in chamber slides, fixed with 2% PFA and immunostained for AnxA2 under non-permeabilized conditions (no Triton-X). Nuclei were stained with DAPI (blue). A single confocal slice is shown in each representative image. Scale bar = 50 μm. **(b)** WT, S100A10 KO, and A2t KO HeLa cells were grown to 80% confluence, washed with heparinase buffer, and incubated with either buffer (vehicle ctrl; left panel) or 2 U/mL heparinase I (removes HSPG; right panel) for 1 h at 37 °C. Cells were transferred to ice, washed with ice-cold PBS, and then saturated with HPV16 pseudovirions (10μg/1E6 cells) in serum-free conditions for 1 h at 4 °C. Cells were collected via scraping on ice, and the amount of surface-bound HPV was analyzed by flow cytometry. Results show the mean fluorescent intensity (MFI) ± s.d., relative to WT HeLa, for three independent replicates (n = 9). Statistics: 2-way ANOVA with Sidak’s multiple comparisons test. ns = not significant.

HPV16 binds to the cell surface through two attachment events. First, through interaction with HSPGs^10–13^ and next, through a secondary receptor or receptor complex at tetraspanin enriched microdomains^9^. Because HPV16 and A2t have been shown to colocalize at the cell surface^25^, we investigated if this interaction is required for either cell surface binding event. To first confirm cell surface AnxA2 localization, we immunolabeled non-permeabilized cells for AnxA2 and analyzed surface expression via immunofluorescent microscopy (Fig. 3a). We observed ubiquitous cell surface expression of AnxA2 in WT and S100A10 KO cells and no expression in A2t KO cells. Cells treated with or without heparinase were incubated with HPV16 at 4 °C to prevent internalization, and the amount of cell surface-bound HPV was quantitated by flow cytometry. The left panel of Fig. 3b shows the amount of HPV16 bound to the cell surface in the presence of HSPG, and there is no statistical difference between WT, S100A10 KO, and A2t KO cells. To examine HPV16 bound to the secondary receptor/receptor complex, HSPGs were removed using heparinase. It is important to note that *in vitro* capsid modifications in lab-produced pseudovirion and virus-like particle systems have been shown to occur at the cell surface after interaction with HSPGs^58,59^. However, furin cleavage is relevant in late-stage intracellular trafficking and is not required for cell surface binding nor internalization^60,61^. Consistent with the reduction reported in HPV-HSPG binding studies, total cell surface-bound HPV16 was reduced by about 60% in all cells treated with heparinase. When comparing KO cells to WT, there was no observable difference in surface-bound HPV16 (Fig. 3b, right panel).

### Bulk HPV internalization is not affected by S100A10 KO and A2t KO, and lysosomal degradation is increased

**Figure 4:**
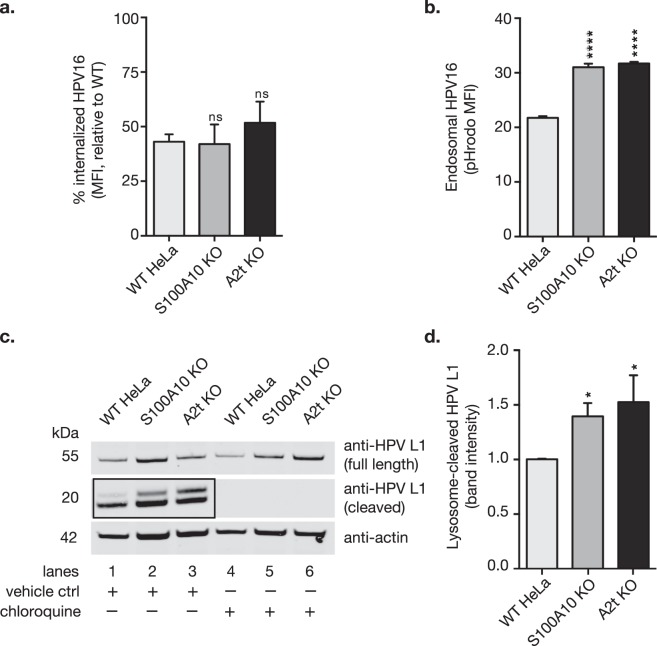
S100A10 and A2t are involved post-HPV entry into HeLa cells, and in the absence of S100A10 and A2t, HPV is redirected to the lysosome and degraded. **(a)** WT, S100A10 KO, and A2t KO cells were treated with HPV16 pseudovirions (PsVs) (5 μg/1E6 cells) for 1 h at 4 °C and measured via flow cytometry. Cells were then incubated for 4 h at 37 °C to promote early internalization, and remaining cell surface-bound HPV was measured via flow cytometry. The difference between the amount of surface-bound HPV at 0 h and 4 h serves as a measure of internalization. Mean fluorescent intensity (MFI) of WT was set to 100% and results show the mean ± s.d. for 3 independent replicates (N = 9). **(b)** pHrodo is a pH-dependent fluorophore that fluoresces in low pH endosomes. Cells were treated with pHrodo-labeled HPV16 (pHrodo-HPV16) VLPs (5 μg/1E6 cells) for 4 h at 37 °C and then measured via flow cytometry. Results show the mean MFI ± s.d., relative to WT HeLa (N = 3), and are representative of at least 3 independent replicates. **(c)** WT, S100A10 KO, and A2t KO cells were pre-treated with either vehicle control or chloroquine, a lysosome acidification inhibitor for 16 h at 37 °C. Cells were then washed on ice and treated with HPV16 pseudovirions (PsVs) (5 μg/1E6 cells) for 1 h at 4 °C, washed, and incubated for 4 h at 37 °C to promote early internalization. Cells were harvested via in-plate lysis and image shows western blot for HPV16 L1 capsid protein (CAMVIR-1 Ab). Full length HPV L1 is visible as a single band (55 kDa), and lysosome-cleaved HPV L1 (~20 kDa) appears as a double band. Western blot is representative of 3 independent experiments. Western blot images were cropped. See full-length blots with molecular weight standards in Supplementary Fig. 1c. **(d)** Quantification of western blot band intensity from 3 independent replicates measured in Image Studio^TM^ Lite. Results show mean band intensity ± s.d. (N = 3, normalized actin and relative to WT). Statistics: 1-way ANOVA with Dunnett’s multiple comparison test – ns = not significant, *P < 0.05, ****P < 0.0001.

We next asked if the observed decrease in infection (Fig. 1d) could be explained by a reduction in HPV internalization in the absence of S100A10 and A2t. By analyzing the abundance of remaining surface-bound virions post-early endocytosis (4 h), we found no difference in HPV16 internalization into WT, S100A10 KO, and A2t KO cells as measured by flow cytometry (Fig. 4a). To determine if virions are trafficked into degradative endosomal compartments, we utilized pHrodo-labeled HPV16 (pHrodo-HPV16) virus-like particles (VLP) to measure virus internalization. pHrodo is a pH-dependent rhodamine dye that fluoresces in low pH environments, such as degradative endosomes. The results show a significant increase in pHrodo-HPV16 signal intensity in both of the KO cell lines compared to WT at 4 hours post-entry (Fig. 4b). Taken together, these data suggest that virions are equally internalized between WT and KO cells, however, HPV16 is preferentially transferred to more acidic pH environments in both S100A10 and A2t KO cells.

Given that the pH of lysosomes is the lowest of the intracellular compartments, we investigated lysosomal degradation of HPV16. When HPV L1 is degraded via proteolytic enzymes in the lysosome, cleaved HPV L1 is visible via western blot as double bands that appear around 20 kDa using the CAMVIR-1 anti-L1 antibody (Fig. 4c, lanes 1–3). Treatment with chloroquine, a lysosome acidification inhibitor, results in complete disappearance of the cleaved HPV L1 bands (Fig. 4c, lanes 4–6). By quantifying the band intensities of the HPV L1 cleavage products normalized to actin in three independent experiments, we confirmed that lysosome cleavage of HPV16 capsids is increased in the absence of S100A10 and A2t (Fig. 4d).

### HPV endocytosis remains clathrin-independent in S100A10 KO and A2t KO cells

**Figure 5:**
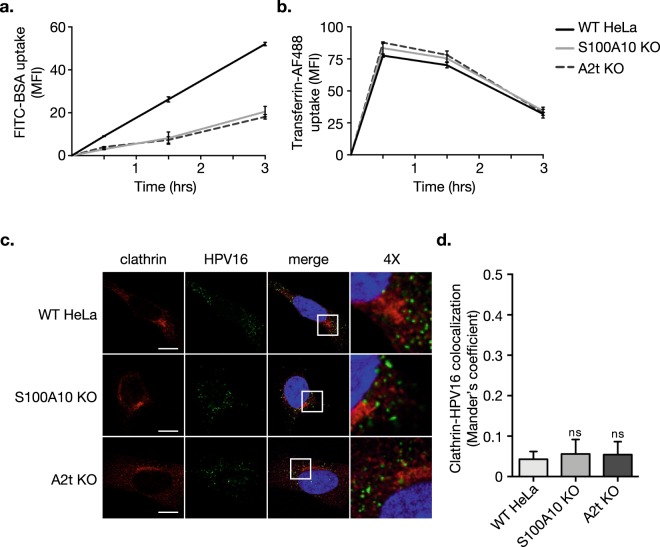
Pinocytosis is reduced, clathrin-mediated endocytosis is unaffected, and HPV endocytosis remains clathrin-independent in S100A10 KO and A2t KO cells. **(a)** Bovine serum albumin (BSA) is taken up via pinocytosis. Here, 10μg/mL of fluorescein isothiocyanate-conjugated BSA (FITC-BSA) was added to pre-cooled WT, S100A10 KO, and A2t KO cells at 4 °C, transferred to 37 °C, and analyzed via flow cytometry at 0.5, 1.5, and 3 h. Results show mean fluorescent intensity (MFI) ± s.d. (N = 3) relative to WT HeLa. **(b)** Transferrin is taken up via clathrin-mediated endocytosis. 10μg/mL of Alexa Flour® 488-conjugated transferrin (transferrin-AF488) was added to cells and analyzed as described in **(a)**. Results show mean fluorescent intensity (MFI) ± s.d. (N = 3) relative to WT HeLa. **(c)** HPV16 endocytosis is shown to be clathrin-independent. HPV16 pseudovirions (0.5 μg/1E6 cells) were added to pre-cooled cells grown on chamber slides, bound for 1 h at 4 °C, washed, and incubated for 1 h at 37 °C. Cells were then fixed with 4% PFA and immunostained for clathrin (red) and HPV16 (green), and nuclei were stained with DAPI (blue). A single confocal slice is shown in each representative image. Scale bar = 10 μm. (**d**) Quantification of extent of clathrin-HPV16 colocalization was measured as Mander’s coefficient using the JACoP plugin for Fiji. Representative results are shown as the mean ± s.d. (N = 11 images). Statistics **(a**,**b**): repeated measures ANOVA (rANOVA) with Dunnett’s multiple comparisons test. Statistics **(d)**: 1-way ANOVA w/ Dunnett’s multiple comparisons test. ns = not significant.

Because AnxA2 and S100A10 have been implicated in multiple membrane trafficking events^62^, we asked if knocking out S100A10 or A2t would have general effects on endocytosis using model endocytic cargos. HPV endocytosis has been shown to be pinocytosis-like^8^, so we first examined the effect of S100A10 KO and A2t KO on pinocytic mechanisms. Fluorescein isothiocyanate-conjugated bovine serum albumin (FITC-BSA) was used as model cargo for pinocytosis^63^, and uptake was analyzed over time. We observed a significant decrease in FITC-BSA uptake in both the S100A10 KO and A2t KO cells compared to WT (Fig. 5a). To verify that clathrin-mediated endocytosis remained intact in the KO cells, we used Alexa Fluor® 488-labeled transferrin (transferrin-AF488) as model cargo for clathrin-mediated endocytosis^64,65^, and analyzed uptake over time. Consistent with the literature, transferrin-AF488 uptake was rapid in WT HeLa cells, and when compared to S100A10 and A2t KO cells, transferrin-AF488 uptake was not affected (Fig. 5b). Finally, to verify that HPV uptake does not get shifted towards clathrin-mediated endocytosis, we used confocal microscopy to analyze the presence of the virus in clathrin-containing compartments (Fig. 5c). Visual and quantitative analysis showed that HPV16 and clathrin do not colocalize in WT, S100A10 KO, and A2t KO cells, as measured by Mander’s coefficient (Fig. 5d).

### A2t controls the progression of HPV-containing early endosomes to late multivesicular endosomes (MVEs), inhibits capsid disassembly, and AnxA2 forms a complex with tetraspanin CD63 during HPV trafficking

**Figure 6:**
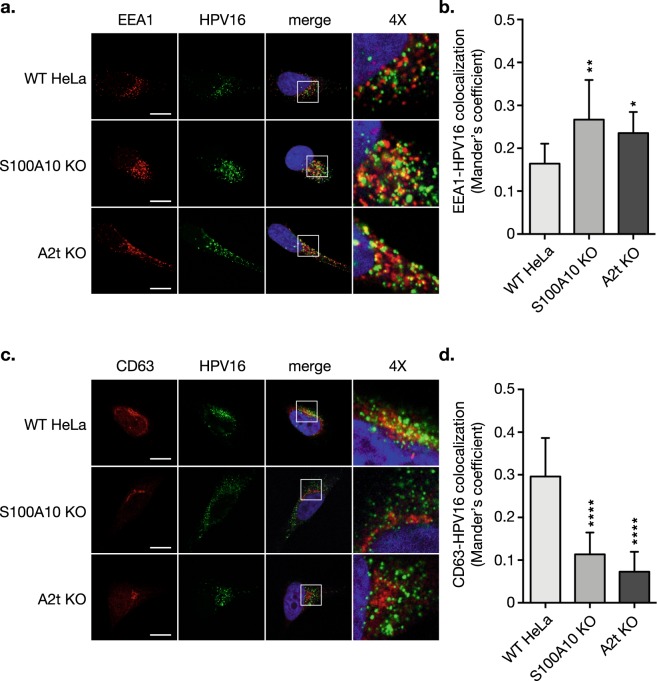
S100A10 knockout (KO) and A2t KO inhibit the progression of HPV16 from early endosomes to multivesicular endosomes (MVEs). After binding to the cell surface, HPV is endocytosed into early endosomes. **(a)** Here, HPV16 pseudovirions (PsVs) (0.5 μg/1E6 cells) were added to pre-cooled cells grown on chamber slides, bound for 1 h at 4 °C, washed, and incubated for 1 h at 37 °C. Cells were then fixed with 4% PFA and immunostained for EEA1 (red) to mark early endosomes, HPV16 (green), and nuclei were stained with DAPI (blue). A single confocal slice is shown in each representative image. Scale bar = 10 μm. **(b)** Quantification of extent of EEA1-HPV16 colocalization was measured as Mander’s coefficient M2 using the JACoP plugin for Fiji. Representative results are shown as the mean ± s.d. (N = 11 images, ≥2 cells/image). **(c)** Post-entry, HPV travels from early endosomes to MVEs, and directly interacts with CD63, a marker for MVEs. Cells were treated as described in **(a)**, incubated for 7 h at 37 °C, fixed with 4% PFA, and immunostained for CD63 (red) and HPV16 (green), and nuclei were stained with DAPI (blue). A single confocal slice is shown in each representative image. Scale bar = 10 μm. **(d)** Quantification of extent of CD63-HPV16 colocalization was measured as Mander’s coefficient using the JACoP plugin for Fiji. Representative results are shown as the mean ± s.d. (N = 10 images). Statistics **(b**,**d**): 1-way ANOVA with Dunnett’s multiple comparisons test – *P < 0.05, **P < 0.01, ****P < 0.0001.

A previous study demonstrated that *in vitro* anti-S100A10 antibody treatment of epithelial cells caused increased colocalization of HPV16 with LAMP1 – a marker of late endosomes/lysosomes, and the authors suggested a role for S100A10 in HPV16 intracellular trafficking^25^. After binding to the cell surface, HPV is endocytosed into early endosomes and then to MVEs in a CD63-dependent fashion^8,34^. We therefore investigated these intracellular trafficking steps in S100A10 KO and A2t KO cells compared to WT. Immunofluorescence microscopy revealed an increase in HPV16 colocalization with early endosome marker EEA1 in KO cells compared to WT (Fig. 6a). Confirming our visual observations, quantification of the extent of red and green overlap showed a significant increase in the extent of HPV16-EEA1 colocalization in the KO cells compared to WT (Fig. 6b). Tetraspanin CD63 is a marker for MVEs, and because CD63 and HPV directly interact, we analyzed the amount of HPV-CD63 colocalization via immunofluorescence microscopy. Visual and quantitative analysis revealed a significant decrease in HPV16-CD63 colocalization (Fig. 6c,d), demonstrating that in the absence of A2t, less HPV16 is shuttled to MVEs when compared to WT cells.

**Figure 7:**
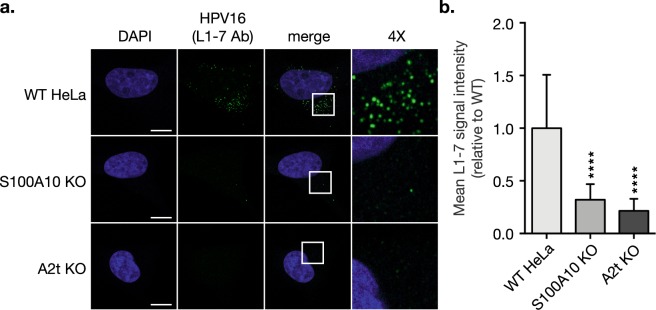
HPV16 capsid disassembly is dramatically inhibited in the absence of S100A10 and A2t. **(a)** WT, S100A10 KO, and A2t KO cells grown on chamber slides were treated with HPV16 pseudovirions (PsVs) (0.5 μg/1E6 cells) for 1 h at 4 °C to promote bulk binding. Cells were then washed and incubated for 7 h at 37 °C to promote endocytosis and intracellular trafficking. Cells were fixed with 4% PFA and immunostained with the anti-HPV 33L1-7 antibody (L1-7 Ab) that recognizes an internal epitope on the HPV capsid (green), marking disassembled capsid. Nuclei were stained with DAPI (blue) and a single confocal slice is shown in each representative image. Scale bar = 10 μm. **(b)** Quantification of L1-7 Ab signal intensity was measured as mean tonal intensity using Fiji. Results are shown as the mean ± s.d. (N = 15 images). Statistics: 1-way ANOVA with Dunnett’s multiple comparisons test – ****P < 0.0001.

HPV capsid disassembly is understood to occur in MVEs, and given that HPV16 abundance in MVEs is significantly decreased in the absence of S100A10 and A2t (Fig. 6c,d), we investigated if capsid disassembly was affected. Using an antibody that specifically recognizes an internal epitope of the HPV capsid (L1-7 Ab), we stained HPV16-treated cells and saw a striking signal reduction in the S100A10 KO and A2t KO cells compared to WT (Fig. 7a), confirmed by quantitative analysis of L1-7 Ab signal (Fig. 7b).

**Figure 8:**
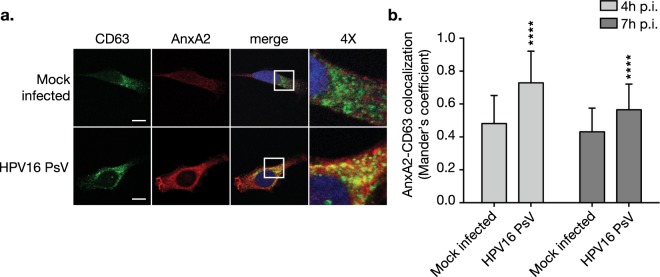
HPV16 infection stimulates CD63-AnxA2 complex formation. **(a)** WT cells grown on chamber slides were treated with HPV16 PsVs (0.5 μg/1E6 cells) for 4 h and 7 h at 37 °C (4 h shown). At 4 h and 7 h post-infection (p.i.) cells were then fixed with 4% PFA and immunostained with the anti-CD63 (green) and anti-AnxA2 (red) antibodies. Nuclei were stained with DAPI (blue) and a single confocal slice is shown in each representative image. Scale bar = 10 μm. **(b)** Quantification of AnxA2-CD63 colocalization was measured as Mander’s coefficient M2 using the JACoP plugin for Fiji. Representative results are shown as the mean ± s.d. (N = 15 images) Statistics: 2-way ANOVA with Sidak’s multiple comparisons test – ****P < 0.0001.

Taken together, these data indicate that failure of infection in the knockouts is due to a blockage of HPV progression from early endosomes to MVEs and subsequent redirection to lysosomes before capsid disassembly can occur. Similar to our results, a study investigating the CD63-syntenin-1 complex in HPV infection found that post-entry delivery of HPV to MVEs and consequent capsid disassembly are dependent on the CD63 complex^34^. Therefore, we hypothesized that AnxA2 and CD63 interact in the context of HPV infection. Immunofluorescence microscopy revealed a significant increase in AnxA2 colocalization with CD63 at 4 h and 7 h post-HPV infection in WT HeLa cells (Fig. 8a,b). The formation of an AnxA2-CD63 complex in HPV infection warrants further investigation, however, these data support a functional role for AnxA2 in HPV intracellular trafficking.

## Discussion

Earlier reports have shown that A2t is required for HPV16 infection of epithelial cells. Our data expand these findings to HPV18, -31, and -45, a subset of genotypes with a high risk for oncogenic transformation^66^, and support a role for A2t in multiple high-risk HPV infections. Because interpreting the role of AnxA2 vs. A2t is unclear in many study designs, we independently knocked out S100A10 and A2t to define the roles of A2t and its subunits in HPV endocytosis. Despite previous evidence demonstrating a direct interaction between S100A10 and HPV L2^24^, inhibition of infection in the S100A10 KO was reproducibly less pronounced compared to the full A2t KO for all genotypes tested. These data strongly suggest that monomeric AnxA2 and heterotetrameric A2t play separable roles in HPV infection, although both KOs showed similar trafficking phenotypes in other experiments. For the additional high-risk genotypes, the reduction in infection in the KO cells is less pronounced compared to HPV16, indicating that although A2t plays an important role, its significance for HPV16 infection cannot be understated. The differences between infection in S100A10 KO versus A2t KO cells could be explained by the fact that S100 family members S100A4, S100A6, and S100A11 are also able to complex with AnxA2 and are expressed in cervical epithelium^67–70^. If other S100-AnxA2 complexes behave similarly to A2t, these alternative binding modes could compensate for the loss of S100A10 and result in the dampened inhibition of HPV infection. Accordingly, AnxA2 re-expression was sufficient to restore HPV16 infection in our rescue experiments, although S100A10 protein levels were not comparable to WT. Alternatively, monomeric AnxA2 may serve an independent function post-endocytic trafficking.

Two primary models for the involvement of A2t in HPV infection have been previously described^24,25^. These models are not mutually exclusive, but A2t’s involvement was studied from two distinct perspectives. The first is based on previous work from our laboratory and emphasizes a direct interaction between the S100A10 subunit of A2t and HPV16 capsid protein L2 (HPV L2), and further posits that A2t is an HPV L2-specific cell surface receptor for HPV16^24,56,71^. The second, based on detailed studies from Ozbun’s laboratory, emphasizes an interaction between AnxA2 and HPV16 capsid protein L1 (HPV L1) at the cell surface, and suggests that S100A10 is involved in post-entry intracellular trafficking^25^. In the absence of S100A10 and A2t, we were surprised to find no difference in the quantity of HPV bound to the cell surface. Given that HPV exposure increases AnxA2 translocation to the cell surface, and because A2t and HPV colocalize at the cell surface^25^, it is reasonable to hypothesize that A2t functions as an HPV receptor. However, we show quantitative evidence that A2t is not a sole requirement for the binding of HPV to the host cell surface. These data do not negate the involvement of A2t at the plasma membrane, but rather indicate that a direct HPV-A2t interaction is not required for cell surface binding. On a larger scale, these data are in agreement with the emerging consensus that HPV cell surface binding involves multi-protein complexes and warrants further work defining an HPV-entry associated membrane platform^72^.

Following HPV binding to the cell surface, we found no significant difference in HPV internalization into S100A10 KO and A2t KO cells compared to WT HeLa cells. These results are inconsistent with previous findings that negative regulation of AnxA2 results in delayed HPV16 internalization^25^. Dziduszko and Ozbun interrogated this pathway using an anti-AnxA2 antibody targeting the N-terminus to inhibit interactions at the surface, and it is difficult to discern whether the observed kinetic effect is due to functional silencing of AnxA2 or antibody-triggered endocytosis. Nonetheless, work from the same study showed an increased colocalization of HPV16 with late endosome/lysosome marker LAMP1 following anti-S100A10 antibody treatment. These data suggested a role for A2t in HPV intracellular trafficking but fell short of giving a complete picture of the discrete post-endocytic steps that A2t and/or its subunits may be regulating. To directly interrogate these differences, we used our KO cells to investigate at which points S100A10 and A2t are required in HPV intracellular trafficking. Our results revealed the same phenotype in the S100A10 and A2t KO cells with respect to HPV binding, endocytosis, and intracellular trafficking events. These findings indicate that the AnxA2 monomer alone is not capable of shuttling the virus to the MVE.

An important feature of this study was analyzing the effect that knocking out S100A10 and A2t has on non-HPV endocytic mechanisms. HPV endocytosis has been shown to be pinocytosis-like, but independent of canonical endocytic pathways such as clathrin-mediated endocytosis, and suggested to be novel^8^. Conversely, some investigators have hypothesized that association of HPV with various cell factors may be indicative of the utilization of multiple entry routes depending on receptor engagement^25^. Our data show that targeting AnxA2 and S100A10 does not lead to aberrant HPV internalization through alternative endocytic pathways, and support the previous hypothesis that HPV employs a specific and potentially novel endocytic pathway. Furthermore, our data directly demonstrate that both the AnxA2 monomer and A2t are involved in pinocytosis but not receptor-mediated, clathrin-dependent endocytosis.

By tracking HPV via confocal microscopy, we found that A2t is required for the progression of HPV from early endosomes to CD63+ MVEs (the late endosome stage for HPV^8,34^), and that monomeric AnxA2 alone is not sufficient to facilitate this process. Subsequently, capsid disassembly was dramatically inhibited in the absence of S100A10 and A2t, confirming previous reports and corroborating our intracellular trafficking results. These results are strikingly similar to those published by Gräßel *et al*. in 2016. In this study, knockdown and knock-in techniques were utilized to demonstrate the requirement of the CD63-syntenin-1 complex in HPV infection and trafficking to MVEs^34^. Not surprisingly, we found that colocalization of AnxA2 and CD63 is increased upon HPV infection. Consistent with observations in the literature, we also observed a notable increase in AnxA2 intensity in HPV-treated cells^25^. Throughout the intracellular trafficking of HPV, AnxA2 and CD63 appear to be members of a complex that likely includes other members (e.g. syntenin-1 and S100A10) and is critical for efficient HPV infection. Taken together, these data suggest that successful infiltration and unchecked descent of HPV into the host nucleus requires A2t-dependent vesicle trafficking from early endosomes to MVEs.

Previous studies have implicated S100A10 in the trafficking of a variety of membrane-resident proteins (reviewed in^41^), but in contradiction to the data supporting these conclusions, another group claims that S100A10 is dispensable for the endosomal transport functions of AnxA2^73,74^. As a result of our study design, we were able to directly test if S100A10 is involved in AnxA2-dependent vesicle trafficking. Utilizing both HPV as model cargo and BSA as pathway-specific canonical cargo in our uptake experiments, we provide clear evidence for a role for S100A10 in AnxA2-associated trafficking. Furthermore, a study investigating MVE biogenesis did not discern between monomeric AnxA2 and A2t by examining the role of S100A10^53^. Our findings support previous work defining a role for AnxA2 and provide the first evidence that heterotetrameric A2t is functioning in MVE biogenesis.

Annexin A2 and its binding partner S100A10 serve dynamic roles in and outside of HPV intracellular trafficking^44,62,75^. The role that AnxA2 and A2t serve at the HPV-host cell surface interface, however, is poorly understood. AnxA2 and A2t are involved in membrane trafficking events including microvesicle formation, vesicle aggregation, phagocytosis, and nucleotide and protein trafficking^76–82^. Notably, these functions are relevant for binding to and co-internalization of HPV with host cell surface receptors and subsequent vesicle trafficking. Although not necessary for cell surface binding, AnxA2 may be required at the cell surface to recruit and organize essential co-factors at the HPV entry platform/receptor complex. In combination with the fact that HPV exposure leads to an increase in cell surface-bound A2t, this is an area that should be further investigated.

This study provides evidence for the involvement of A2t in HPV trafficking from early endosomes to MVEs in a CD63-dependent fashion, protection from lysosomal degradation, and that A2t is requisite for HPV capsid disassembly. Furthermore, using HPV as a model, we provide evidence that S100A10 is involved in AnxA2-mediated endosomal transport and MVE biogenesis. By implementing methods that can differentiate between AnxA2- and A2t-dependent effects in HPV intracellular trafficking, we solidify existing knowledge about AnxA2-dependent endocytosis, and provide a framework for future studies investigating the role of AnxA2 vs. A2t in non-HPV endocytosis through analysis of S100A10-associated functions.

## Methods

### Cell culture, CRISPR/Cas9 gene editing, and clone proliferation analysis

HeLa cells (CCL-2, ATCC) derived from cervical carcinoma were maintained in Iscove’s Modified Dulbecco’s Medium (IMDM) with 10% FBS, 1x BME, and 1x gentamycin at 37 °C with 5% CO_2_.

Addgene plasmid pX335-U6-Chimeric_BB-CBh-hSpCas9n(D10A) (#42335; a gift from Ling Shao) was used for CRISPR/Cas9-targeted ANXA2 and S100A10 gene disruption via mutant Cas9 nickase (D10A variant). The following target sequences were used: 5′-CAGGAAGCACGAACATCAGC-3′ and 5′-ACAGGGGCTGGGAACCGACG-3′ for ANXA2; and 5′-AATGGTGAGGCCCGCAATTA-3′ and 5′-GTAGTACACATGAAGCAGAA-3′ for S100A10. Guide RNA (gRNA) were cloned using Feng Zhang Laboratory protocols^83^. Non-target plasmid was used as negative control and pMSCVpuro vector (a gift from Ling Shao) was used for selection. HeLa cells co-transfected with Lipofectamine2000 (Thermo Fisher) were selected with puromycin for 72 h, and clonal populations were generated through a limited dilution series. Clonal populations were then sequenced to verify gene disruption and analyzed for protein expression via western blot.

Knockout clones were analyzed for differences in cell proliferation via cell counting (trypan blue exclusion method) as well as DNA quantification using the CyQUANT Cell Proliferation Assay Kit (Invitrogen). Briefly, WT, S100A10 KO, and A2t KO cells were grown for 48 h, collected with trypsin-EDTA, diluted 1:1 with trypan blue stain (Invitrogen), and viable cells were counted. To quantify DNA via CyQUANT kit, cells were cultured for 48 h, frozen at −80 °C overnight, thawed at room temperature, and lysed using CyQUANT cell lysis buffer with RNAse and GR dye according to manufacturer protocol. Fluorescence of GR dye-bound DNA was measured using a SpectraMax M5 plate reader (Molecular Devices) and DNA was quantified by comparison to DNA standard curve.

### Pseudovirion and virus-like particle production

HPV16, -18, -31, and -45 PsVs were prepared as previously described^84^ (http://home.ccr.cancer.gov/LCO/ripcord.htm). Briefly, 293TT cells were co-transfected with codon-optimized L1 and L2 plasmids for HPV16, -18, -31, and -45, and pCIneoGFP reporter plasmid. For bulk PsV preparations, the self-packaging p16L1L2 plasmid was utilized. HPV L1 and L2 expression vectors used were p16L1L2 (bulk), p16sheLL, p18sheLL, p31sheLL, and p45sheLL (all kind gifts from J. Schiller). Infectious titer was determined by flow cytometric analysis of GFP+ 293TT cells 48 h post-treatment and calculated as infectious units/mL. Bulk PsV preps were quantified via coomassie blue staining utilizing known BSA concentrations.

HPV16 virus-like particles (VLPs) were produced using a recombinant baculovirus expression system in insect cells as previous described^85^. Western blot analysis confirmed the presence of HPV L1 and L2, while a neutralizing antibody ELISA confirmed the presence of intact particles. Coomassie Blue staining was performed to determine protein purity and standardize the concentration of HPV L1 content of the VLP preparations.

### Antibodies

Previously described anti-HPV16 L1-specific neutralizing antibody H16.V5 and internal epitope-recognizing 33L1-7 (L1-7 Ab) were gifts from Neil Christensen and Martin Sapp, respectively^86,87^. Anti-HPV16 L1 (H16.56E) used in immunofluorescence experiments was a gift from Martin Sapp^88^. Anti-AnxA2 (610069), anti-S100A10 (610071), and anti-HPV16 L1 (550840) were purchased from BD Biosciences. Mouse IgG isotype control (ab37355), rabbit IgG isotype control (ab37415), anti-CD63 (ab118307), and goat-anti-rabbit TRITC (ab6718) were purchased from Abcam. Anti-β actin (4970), anti-clathrin (4796), and anti-EEA1 (3288) were purchased from Cell Signaling Technology. Anti-mCherry (677702) and goat-anti-mouse DyLight 488 (405310) were purchased from BioLegend. Antibodies used in AnxA2-CD63 colocalization immunofluorescence microscopy were anti-AnxA2 (11256-1-AP) and anti-CD63 (ab8219) from Proteintech and Abcam, respectively. Goat-anti-mouse IRDye 800CW (925-322) and goat-anti-rabbit (H + L) Alexa Fluor 680 (A27042) secondary antibodies used for infrared western blot imaging were purchased from Li-Cor and Invitrogen, respectively.

### Pseudovirus infection and infection rescue assay

2E4 cells were seeded in 24-well plates and infected with a 50% tissue culture infective dose (TCID_50_) the following day. TCID50 was determined by titrating the multiplicity of infection (MOI) for each genotype to result in approximately 50% infected cells, 48 h post-infection. Infection is defined in this manuscript as gene transduction of the GFP reporter plasmid. In infection assays, %GFP-positive cells were analyzed 48 h post-virus addition via flow cytometry (FC500, Beckman Coulter). For rescue experiments, cells were seeded in 6-well plates, grown to 80% confluency, and transfected with pEGFP-N3_ANXA2-mCherry (a gift from Volker Gerke) or an empty vector using Lipofectamine 2000 (Thermo Fisher). Utilizing HPV16, cells were subjected to the HPV-infectivity assay as described above and infection rescue was analyzed by first gating mCherry-positive cells and then measuring %GFP-positive cells within that population using CXP software (version 2.2) (Supplementary Fig. 2).

### Cell surface binding assay

2.5E5 cells were seeded in 6-well plates and grown overnight. Cells were washed with ice cold buffer (20 mM Tris-HCl, 50 mM NaCl, 4 mM CaCl_2_, and 0.01% BSA) and then treated with either buffer (vehicle control) or 2 U/mL heparinase I (Sigma-Aldrich) for 1 h at 37 °C to remove HSPGs. Plates were transferred to ice and washed thoroughly with ice cold PBS + CaCl_2_. PBS supplemented with 1 mM CaCl_2_ was used for all wash steps as AnxA2 binds membranes in a calcium-dependent manner. Cells were then treated with 10 μg/1E6 cells HPV16 in cold serum-free media for 1 h at 4 °C to saturate binding. Cells were collected on ice via scraping, stained with H16.V5 (1:100) for 30 min at 4 °C, and fixed with 2% PFA in order to ensure measurement of particles that might internalize at room temperature during analysis. Mean fluorescent intensity (MFI) was measured via flow cytometry.

### Virus internalization assays

2.5E5 cells were seeded in 6-well plates and grown overnight. Plates were cooled to 4 °C for 30 min and 5 μg/1E6 cells of HPV16 PsVs were added for 1 hr at 4 °C. Cells were collected and surface-bound HPV was measured as described in cell surface binding assay. Unbound virus was washed away with PBS + CaCl_2_ and cells were incubated for an additional 4 h at 37 °C to promote internalization of surface-bound HPV. After 4 h, remaining surface-bound HPV was again analyzed via flow cytometry. The amount of internalized PsV was determined by subtracting MFI at 4 h (remaining surface HPV) from MFI at 0 h (initial surface HPV). MFI of WT HeLa cells was set to 100% and KO cells were normalized to that value. For pHrodo-HPV16 uptake assays, HPV16 VLPs were labeled with pH-dependent rhodamine fluorophore (pHrodo iFL Red STP, Life Technologies) in a 10:1 (dye:HPV L1 protein) ratio, purified with 2% agarose beads (sized 50–150 µm) (Gold Biotechnology), and treated as described above. Briefly, after 30 min at 4 °C cells were treated with HPV16 PsVs for 4 h at 37 °C and MFI of pHrodo-HPV16 was measured via flow cytometry. MFI was measured from 0–12 h and trends remained consistent.

### Lysosome degradation assay via Western blot

2.5E5 cells were seeded in 6-well plates, allowed to adhere, and then treated overnight (16 h) with 50 μM chloroquine diphosphate crystalline (Sigma-Aldrich) or vehicle control. Plates were cooled to 4 °C for 30 min, treated with 5 μg/1E6 cells HPV16 PsVs for 1 hr at 4 °C, thoroughly washed with PBS + CaCl_2_, and then incubated for 4 h at 37 °C to promote internalization. Cells were collected by in-plate lysis using M-PER Mammalian Protein Extraction Reagent (Thermo Fisher) and lysates were analyzed via western blot for cleaved HPV L1 (CAMVIR-1 antibody) and β-actin using Odyssey Infrared blot imager (Li-Cor).

### Endocytic uptake assays

2.5E5 cells were seeded in 6-well plates and grown overnight. Plates were cooled to 4 °C for 30 min and either Alexa Fluor 488-conjugated transferrin from human serum (Transferrin-AF488, Thermo Fisher) or fluorescein isothiocyanate (FITC)-conjugated bovine serum albumin (BSA) (FITC-BSA, Sigma-Aldrich) were added, and cells were transferred to 37 °C. Cells were collected at 0.5, 1.5, and 3 h after the temperature shift, and MFI was measured via flow cytometry.

### Immunofluorescence microscopy

For HPV colocalization with EEA1 and CD63, and for L1-7 Ab staining experiments, cells were seeded on 8-well chamber slides (1E4 cell/well) and grown overnight. Slides were cooled to 4 °C for 30 min prior to treatment with 0.5 μg/1E6 cells of HPV16 PsVs for 1 h or 7 h at 37 °C. At 1 h, cells were stained for HPV16 (H16.56E, 1:100) and either EEA1 (1:100) or clathrin (1:50). At 7 h, cells were stained for HPV16 (H16.56E) and either CD63 (1:100), or L1-7 Ab (1:10). For AnxA2-CD63 colocalization, cells were seeded on 3-well chamber slides (2.5E4 cells/well) and grown overnight. Slides were treated with HPV16 PsVs at 0.5 μg/1E6 cells for 4 h or 7 h at 37 °C. Cells were stained for AnxA2 (1:100) and CD63 (1:100). For imaging in non-permeabilized conditions, Triton X-100 was omitted from the immunostaining procedure. All slides were fixed with 2–4% PFA prior to staining and mounted with ProLong Gold Antifade Mountant with DAPI (Thermo Fisher). Images were visualized on a Nikon Eclipse Ti-E laser scanning confocal microscope. For colocalization analysis, 10–20 images were analyzed with Fiji (a distribution of ImageJ, NIH)^89,90^. Using the JACoP plugin^91^, thresholds were automatically set, and the extent of colocalization was measured using Mander’s colocalization coefficient. Batch image analysis was achieved through an application-specific script in Fiji macro language.

### Statistics

Background from control groups was subtracted in all experiments and KO cells were normalized to WT cells for comparison. Statistical analyses were performed using GraphPad Prism (version 6) and detailed statistical tests for each figure are provided in legends.

### Data availability

The datasets generated during the current study are available from the corresponding author upon reasonable request.

## Electronic supplementary material

Supplementary Figures 1 and 2

## References

[1] Forman D (2012). Global Burden of Human Papillomavirus and Related Diseases. Vaccine.

[2] Watson M (2008). Using population-based cancer registry data to assess the burden of human papillomavirus-associated cancers in the United States: Overview of methods. Cancer.

[3] Spence T, Bruce J, Yip KW, Liu FF (2016). HPV associated head and neck cancer. Cancers (Basel)..

[4] Zheng Z-M, Baker CC (2006). Papillomavirus genome structure, expression, and post-transcriptional regulation. Front. Biosci..

[5] Qi YM (1996). Epithelial cells display separate receptors for papillomavirus VLPs and for soluble L1 capsid protein. Virology.

[6] DiGiuseppe S, Bienkowska-Haba M, Guion LG, Sapp M (2017). Cruising the cellular highways: How human papillomavirus travels from the surface to the nucleus. Virus Res..

[7] Fausch SC, Da Silva DM, Kast WM (2003). Differential uptake and cross-presentation of human papillomavirus virus-like particles by dendritic cells and Langerhans cells. Cancer Res..

[8] Schelhaas, M. *et al*. Entry of human papillomavirus type 16 by actin-dependent, clathrin- and lipid raft-independent endocytosis. *PLoS Pathog*. **8** (2012). 10.1371/journal.ppat.1002657 PMC3334892 22536154

[9] Spoden, G. *et al*. Clathrin- and caveolin-independent entry of human papillomavirus type 16 - Involvement of tetraspanin-enriched microdomains (TEMs). *PLoS One* **3** (2008). 10.1371/journal.pone.0003313 PMC2561052 18836553

[10] Giroglou T, Florin L, Schafer F, Streeck RE, Sapp M (2001). Human Papillomavirus Infection Requires Cell Surface Heparan Sulfate. J. Virol..

[11] Selinka H-C (2007). Inhibition of Transfer to Secondary Receptors by Heparan Sulfate-Binding Drug or Antibody Induces Noninfectious Uptake of Human Papillomavirus. J. Virol..

[12] Johnson KM (2009). Role of Heparan Sulfate in Attachment to and Infection of the Murine Female Genital Tract by Human Papillomavirus. J. Virol..

[13] Kines RC, Thompson CD, Lowy DR, Schiller JT, Day PM (2009). The initial steps leading to papillomavirus infection occur on the basement membrane prior to cell surface binding. Proc. Natl. Acad. Sci..

[14] Selinka H-C, Giroglou T, Nowak T, Christensen ND, Sapp M (2003). Further evidence that papillomavirus capsids exist in two distinct conformations. J. Virol..

[15] Dasgupta J (2011). Structural basis of oligosaccharide receptor recognition by human papillomavirus. J. Biol. Chem..

[16] Richards KF, Bienkowska-Haba M, Dasgupta J, Chen XS, Sapp M (2013). Multiple Heparan Sulfate Binding Site Engagements Are Required for the Infectious Entry of Human Papillomavirus Type 16. J. Virol..

[17] Bienkowska-Haba, M., Patel, H. D. & Sapp, M. Target cell cyclophilins facilitate human papillomavirus type 16 infection. *PLoS Pathog*. **5** (2009). 10.1371/journal.ppat.1000524 PMC2709439 19629175

[18] Scheffer KD (2013). Tetraspanin CD151 Mediates Papillomavirus Type 16 Endocytosis. J. Virol..

[19] Scheffer KD, Berditchevski F, Florin L (2014). The tetraspanin CD151 in papillomavirus infection. Viruses.

[20] Evander M (1997). Identification of the alpha6 integrin as a candidate receptor for papillomaviruses. J. Virol..

[21] McMillan NAJ, Payne E, Frazer IH, Evander M (1999). Expression of the α6 integrin confers papillomavirus binding upon receptor-negative B-cells. Virology.

[22] Surviladze, Z., Dziduszko, A. & Ozbun, M. A. Essential roles for soluble virion-associated heparan sulfonated proteoglycans and growth factors in human papillomavirus infections. *PLoS Pathog*. **8** (2012). 10.1371/journal.ppat.1002519 PMC3276557 22346752

[23] Surviladze Z, Sterk RT, DeHaro SA, Ozbun MA (2013). Cellular Entry of Human Papillomavirus Type 16 Involves Activation of the Phosphatidylinositol 3-Kinase/Akt/mTOR Pathway and Inhibition of Autophagy. J. Virol..

[24] Woodham, A. W. *et al*. The S100A10 subunit of the annexin A2 heterotetramer facilitates L2-mediated human papillomavirus infection. *PLoS One* **7** (2012). 10.1371/journal.pone.0043519 PMC3425544 22927980

[25] Dziduszko A, Ozbun MA (2013). Annexin A2 and S100A10 Regulate Human Papillomavirus Type 16 Entry and Intracellular Trafficking in Human Keratinocytes. J. Virol..

[26] Broniarczyk J, Massimi P, Bergant M, Banks L (2015). Human Papillomavirus Infectious Entry and Trafficking Is a Rapid Process. J. Virol..

[27] Becker, M., Greune, L., Schmidt, M. A. & Schelhaas, M. Extracellular conformational changes in the capsid of human papillomaviruses contribute to asynchronous uptake into host cells. *J*. *Virol*., 10.1128/JVI.02106-17 (2018). 10.1128/JVI.02106-17 PMC5952151 29593032

[28] Broniarczyk J, Bergant M, Goździcka-Józefiak A, Banks L (2014). Human papillomavirus infection requires the TSG101 component of the ESCRT machinery. Virology.

[29] Broniarczyk J (2017). The VPS4 component of the ESCRT machinery plays an essential role in HPV infectious entry and capsid disassembly. Sci. Rep..

[30] Bergant M, Banks L (2013). SNX17 Facilitates Infection with Diverse Papillomavirus Types. J. Virol..

[31] Wüstenhagen E (2016). The cytoskeletal adaptor obscurin-like 1 interacts with the HPV16 capsid protein L2 and is required for HPV16 endocytosis. J. Virol..

[32] Selinka HC, Giroglou T, Sapp M (2002). Analysis of the infectious entry pathway of human papillomavirus type 33 pseudovirions. Virology.

[33] Bienkowska-Haba M, Williams C, Kim SM, Garcea RL, Sapp M (2012). Cyclophilins Facilitate Dissociation of the Human Papillomavirus Type 16 Capsid Protein L1 from the L2/DNA Complex following Virus Entry. J. Virol..

[34] Gräßel L (2016). The CD63-Syntenin-1 Complex Controls Post-Endocytic Trafficking of Oncogenic Human Papillomaviruses. Sci. Rep..

[35] Day PM, Thompson CD, Schowalter RM, Lowy DR, Schiller JT (2013). Identification of a Role for the trans-Golgi Network in Human Papillomavirus 16 Pseudovirus Infection. J. Virol..

[36] Lipovsky A (2013). Genome-wide siRNA screen identifies the retromer as a cellular entry factor for human papillomavirus. Proc. Natl. Acad. Sci..

[37] Bienkowska-Haba M (2017). Incoming human papillomavirus 16 genome is lost in PML protein-deficient HaCaT keratinocytes. Cell. Microbiol..

[38] Pyeon, D., Pearce, S. M., Lank, S. M., Ahlquist, P. & Lambert, P. F. Establishment of human papillomavirus infection requires cell cycle progression. *PLoS Pathog*. **5** (2009). 10.1371/journal.ppat.1000318 PMC2642596 19247434

[39] Aydin, I. *et al*. Large Scale RNAi Reveals the Requirement of Nuclear Envelope Breakdown for Nuclear Import of Human Papillomaviruses. *PLoS Pathog*. **10** (2014). 10.1371/journal.ppat.1004162 PMC4038628 24874089

[40] Waisman DMA (1995). tetramer: structure and function. Mol. Cell. Biochem..

[41] Rescher U, Gerke V (2008). S100A10/p11: Family, friends and functions. Pflugers Arch. Eur. J. Physiol..

[42] Hitchcock JK, Katz AA, Schäfer G (2014). Dynamic reciprocity: The role of annexin A2 in tissue integrity. J. Cell Commun. Signal..

[43] Hedhli, N. *et al*. The annexin A2/S100A10 system in health and disease: Emerging paradigms. *J*. *Biomed*. *Biotechnol*. 2012 (2012). 10.1155/2012/406273 PMC3496855 23193360

[44] Hajjar KA (2015). The Biology of Annexin A2: From Vascular Fibrinolysis to Innate Immunity. Trans. Am. Clin. Climatol. Assoc..

[45] Schloer S, Pajonczyk D, Rescher U (2018). Annexins in Translational Research: Hidden Treasures to Be Found. Int. J. Mol. Sci..

[46] Pena-Alonso E (2008). Annexin A2 localizes to the basal epithelial layer and is down-regulated in dysplasia and head and neck squamous cell carcinoma. Cancer Lett..

[47] Munz, B., Gerke, V., Gillitzer, R. & Werner, S. Differential expression of the calpactin I subunits annexin II and p11 in cultured keratinocytes and during wound repair. *J*. *Invest*. *Dermatol*. **108**, 307–312 (1997). 10.1111/1523-1747.ep12286470 9036930

[48] Menke M, Gerke V, Steinem C (2005). Phosphatidylserine membrane domain clustering induced by annexin A2/S100A10 heterotetramer. Biochemistry.

[49] Drücker P, Pejic M, Galla HJ, Gerke V (2013). Lipid segregation and membrane budding induced by the peripheral membrane binding protein annexin A2. J. Biol. Chem..

[50] Wayne, N. & Waismanj, M. Calcium-dependent by Lipocortin-85″ Regulation of Actin Filament Bundling. 265, 3392–3401 (1990). 2137457

[51] Hayes MJ, Shao D, Bailly M, Moss SE (2006). Regulation of actin dynamics by annexin 2. EMBO J..

[52] Bharadwaj, A., Bydoun, M., Holloway, R. & Waisman, D. *Annexin A2 heterotetramer: Structure and function*. *International Journal of Molecular Sciences* **14** (2013). 10.3390/ijms14036259 PMC3634455 23519104

[53] Mayran N, Parton RG, Gruenberg J (2003). Annexin II regulates multivesicular endosome biogenesis in the degradation pathway of animal cells. EMBO J..

[54] Puisieux, A., Ji, J. & Ozturk, M. Annexin II up-regulates cellular levels of p11 protein by a post-translational mechanisms. *Biochem J* **313** Pt 1, 51–55 (1996). 10.1042/bj3130051 PMC1216908 8546709

[55] He KL (2008). Endothelial cell annexin A2 regulates polyubiquitination and degradation of its binding partner S100A10/p11. J. Biol. Chem..

[56] Woodham, A. W. *et al*. Small molecule inhibitors of the annexin A2 heterotetramer prevent human papillomavirus type 16 infection. *J*. *Antimicrob*. *Chemother*. 1686–1690, 10.1093/jac/dkv045 (2015). 10.1093/jac/dkv045 PMC4447890 25712315

[57] Benaud C (2015). Annexin A2 is required for the early steps of cytokinesis. EMBO Rep..

[58] Buck CB (2006). Human alpha-defensins block papillomavirus infection. Proc. Natl. Acad. Sci. USA.

[59] Calton CM (2017). Translocation of the papillomavirus L2/vDNA complex across the limiting membrane requires the onset of mitosis. PLoS Pathog..

[60] Richards RM, Lowy DR, Schiller JT, Day PM (2006). Cleavage of the papillomavirus minor capsid protein, L2, at a furin consensus site is necessary for infection. Proc. Natl. Acad. Sci. USA.

[61] Wiens ME, Smith JG (2015). Alpha-Defensin HD5 Inhibits Furin Cleavage of Human Papillomavirus 16 L2 To Block Infection. J. Virol..

[62] Rescher U (2004). Annexins - unique membrane binding proteins with diverse functions. J. Cell Sci..

[63] Kenisi H (2004). Cell surface-expressed phosphatidylserine and annexin A5 open a novel portal of cell entry. J. Biol. Chem..

[64] Conrad ME, Umbreit JN (2000). Iron absorption and transport-an update. Am. J. Hematol..

[65] Conner SD, Schmid SL (2003). Regulated portals of enrty into the cell. Nature.

[66] Ramanakumar AV (2010). Human papillomavirus (HPV) types 16, 18, 31, 45 DNA loads and HPV-16 integration in persistent and transient infections in young women. BMC Infect. Dis..

[67] Semov A (2005). Metastasis-associated protein S100A4 induces angiogenesis through interaction with annexin II and accelerated plasmin formation. J. Biol. Chem..

[68] Nedjadi T (2009). S100A6 binds to annexin 2 in pancreatic cancer cells and promotes pancreatic cancer cell motility. Br. J. Cancer.

[69] Rintala-Dempsey AC, Santamaria-Kisiel L, Liao Y, Lajoie G, Shaw GS (2006). Insights into S100 target specificity examined by a new interaction between S100A11 and annexin A2. Biochemistry.

[70] Rintala-Dempsey AC, Rezvanpour A, Shaw GS (2008). S100-annexin complexes - Structural insights. FEBS J..

[71] Raff AB (2013). The Evolving Field of Human Papillomavirus Receptor Research: a Review of Binding and Entry. J. Virol..

[72] Aksoy P, Gottschalk EY, Meneses PI (2017). HPV entry into cells. Mutat. Res. - Rev. Mutat. Res..

[73] Morel, E. & Gruenberg, J. The p11/S100A10 light chain of annexin A2 is dispensable for annexin A2 association to endosomes and functions in endosomal transport. *PLoS One* **2**, (2007). 10.1371/journal.pone.0001118 PMC2040519 17971878

[74] Morel E, Parton RG, Gruenberg J (2009). Annexin A2-Dependent Polymerization of Actin Mediates Endosome Biogenesis. Dev. Cell.

[75] Gerke V, Moss SE (2002). [LIDO/COMPLEXO] Annexins: From Structure to Function. Physiol. Rev.

[76] Wang S, Sun H, Tanowitz M, Liang XH, Crooke ST (2016). Annexin A2 facilitates endocytic trafficking of antisense oligonucleotides. Nucleic Acids Res..

[77] Grewal T (2009). & Enrich, C. Annexins - Modulators of EGF receptor signalling and trafficking. Cell. Signal..

[78] Zhang, W. *et al*. Annexin A2 Promotes the Migration and Invasion of Human Hepatocellular Carcinoma Cells *In Vitro* by Regulating the Shedding of CD147-Harboring Microvesicles from Tumor Cells. *PLoS One* **8** (2013). 10.1371/journal.pone.0067268 PMC3741296 23950866

[79] Stukes S (2016). The Membrane Phospholipid Binding Protein Annexin A2 Promotes Phagocytosis and Nonlytic Exocytosis of *Cryptococcus neoformans* and Impacts Survival in Fungal Infection. J. Immunol..

[80] Tcatchoff, L. *et al*. Annexin A1 and A2: Roles in retrograde trafficking of Shiga toxin. *PLoS One* **7** (2012). 10.1371/journal.pone.0040429 PMC3391278 22792315

[81] Johnstone SA, Hubaishy I, Waisman DM (1992). Phosphorylation of annexin II tetramer by protein kinase C inhibits aggregation of lipid vesicles by the protein. J. Biol. Chem..

[82] Dathe C (2014). Annexin A2 mediates apical trafficking of renal Na + −K+−2Cl- Cotransporter. J. Biol. Chem..

[83] Cong L (2013). Multiplex Genome Engineering Using CRISPR/VCas Systems. Science (80-.)..

[84] Buck, C. B. & Thompson, C. D. Production of Papillomavirus-Based Gene Transfer Vectors. *Curr*. *Protoc*. *Cell Biol*. 1–19 (2007). 10.1002/0471143030.cb2601s37 10.1002/0471143030.cb2601s37 18228512

[85] Kirnbauer R, Booyt F, Chengt N, Lowy DR, Schiller JT (1992). Papillomavirus Li major capsid protein self-assembles into virus-like particles that are highly immunogenic. Med. Sci..

[86] Christensen ND (1996). Surface conformational and linear epitopes on HPV-16 and HPV-18 L1 virus-like particles as defined by monoclonal antibodies. Virology.

[87] Sapp M (1994). Analysis of type-restricted and cross-reactive epitopes on virus- like particles of human papillomavirus type 33 and in infected tissues using monoclonal antibodies to the major capsid protein. J. Gen. Virol..

[88] Rommel O (2005). Heparan sulfate proteoglycans interact exclusively with conformationally intact HPV L1 assemblies: Basis for a virus-like particle ELISA. J. Med. Virol..

[89] Schindelin J, Rueden CT, Hiner MC, Eliceiri KW (2015). The ImageJ ecosystem: An open platform for biomedical image analysis. Mol. Reprod. Dev..

[90] Schindelin J (2012). Fiji: An open-source platform for biological-image analysis. Nat. Methods.

[91] Bolte S, Cordelieres FP (2006). A guided tour into subcellular colocalisation analysis in light microscopy. J. Microsc..

