# Supplementary material for: Heterotetrameric annexin A2/S100A10 (A2t) is essential for oncogenic human papillomavirus trafficking and capsid disassembly, and protects virions from lysosomal degradation
Source: Sci Rep. 2018 Aug 3;8:11642. doi: 10.1038/s41598-018-30051-2 (PMC6076308; doi:10.1038/s41598-018-30051-2)
Supplement: Supplementary file 1 — Supplementary Figures 1 and 2 [file 41598_2018_30051_MOESM1_ESM.pdf]

Heterotetrameric annexin A2/S100A10 (A2t) is essential for oncogenic human papillomavirus trafficking and capsid disassembly, and protects virions from lysosomal degradation

Julia R. Taylor[1], Daniel J. Fernandez[1], Shantaé M. Thornton[1], Joseph G. Skeate[1], Kim P. Lühen[3], Diane M. Da Silva[2,3], Ralf Langen[4], and W. Martin Kast[1,2,3*]

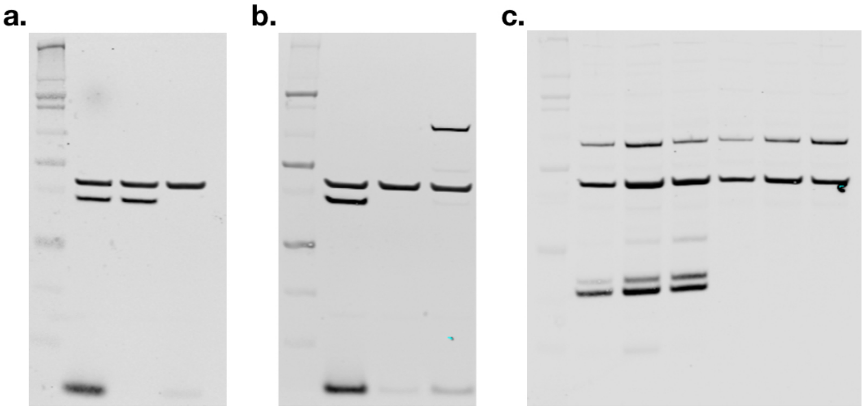

Supplementary Figure 1. Full-length immunoblots with molecular weight standards for **(a)** Figure 1a, **(b)** Figure 2a, and **(c)** Figure 4c.

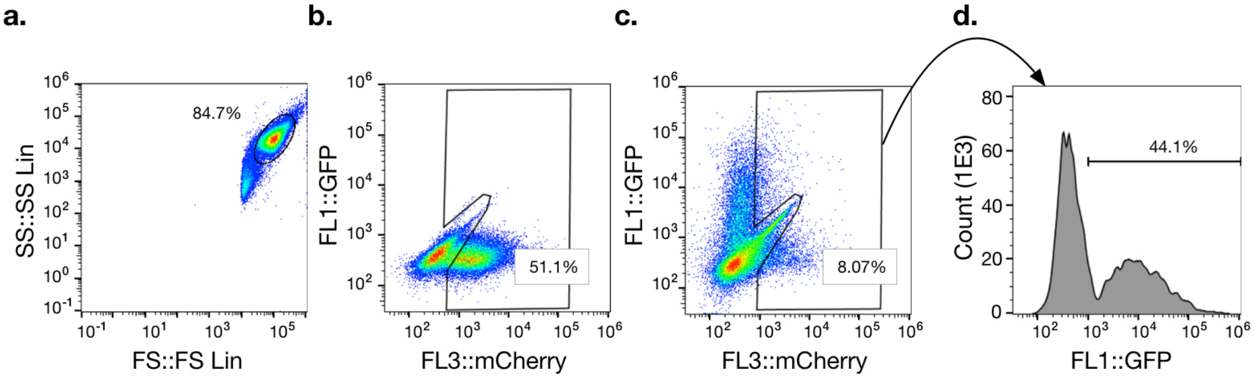

Supplementary Figure 2. Gating strategy for analysis of infection rescue (Fig. 2b). Live cells were gated on forward and side scatter in all plots. **(a)** Control cell population: 50,000 total cells analyzed, 42,350 live cells. **(b)** A2t knockout (KO) cells, 24h post-AnxA2-mCherry transfection: 50,000 total cells analyzed, 37,502 live cells, and 19,173 live mCherry-positive cells. **(c)** A2t KO cells 72h post-AnxA2-mCherry transfection, 48h post-infection: 50,000 total cells analyzed, 43,861 live cells, 3,701 live mCherry-positive cells. **(d)** A2t KO cells 72h post-AnxA2-mCherry transfection, 48h post-infection: 50,000 total cells analyzed, 43,861 live cells, 3,701 live mCherry-positive cells, 1,633 live mCherry-positive GFP-positive cells.
